# Performance and workflow assessment of six nucleic acid extraction technologies for use in resource limited settings

**DOI:** 10.1371/journal.pone.0215753

**Published:** 2019-04-18

**Authors:** Shivani G. Beall, Jason Cantera, Maureen H. Diaz, Jonas M. Winchell, Lorraine Lillis, Heather White, Michael Kalnoky, James Gallarda, David S. Boyle

**Affiliations:** 1 Centers for Disease Control and Prevention, Division of Bacterial Diseases, Respiratory Diseases Branch, Atlanta, Georgia, United States of America; 2 PATH, Seattle, Washington, United States of America; 3 Bill and Melinda Gates Foundation, Seattle, Washington, United States of America; University of Helsinki, FINLAND

## Abstract

Infectious disease nucleic acid amplification technologies (NAAT) have superior sensitivity, specificity, and rapid time to result compared to traditional microbiological methods. Recovery of concentrated, high quality pathogen nucleic acid (NA) from complex specimen matrices is required for optimal performance of several NA amplification/detection technologies such as polymerase chain reaction (PCR). Fully integrated NAAT platforms that enable rapid sample-to-result workflows with minimal user input are generally restricted to larger reference lab settings, and their complexity and cost are prohibitive to widespread implementation in resource limited settings (RLS). Identification of component technologies for incorporation of reliable and affordable sample preparation with pathogen NA amplification/detection into an integrated platform suitable for RLS, is a necessary first step toward achieving the overarching goal of reducing infectious disease-associated morbidity and mortality globally. In the current study, we evaluate the performance of six novel NA extraction technologies from different developers using blinded panels of stool, sputum and blood spiked with variable amounts of quality-controlled DNA- and/or RNA-based microbes. The extraction efficiencies were semi-quantitatively assessed using validated real-time reverse transcription (RT)-PCR assays specific for each microbe and comparing target-specific RT-PCR results to those obtained with reference NA extraction methods. The technologies were ranked based on overall diagnostic accuracy (analytical sensitivity and specificity). Sample input and output volumes, total processing time, user-required manual steps and cost estimates were also examined for suitability in RLS. Together with the performance analysis, these metrics were used to select the more suitable candidate technologies for further optimization of integrated NA amplification and detection technologies for RLS.

## Introduction

An accurate and rapid diagnostic test can facilitate better patient care by informing prompt and appropriate treatment, leading to decreased morbidity and mortality while saving money and preventing improper use of antimicrobial drugs [1]. Rapid diagnostics are also critical to identify pathogens for which targeted public health interventions can be employed to control disease transmission in the community. Nucleic acid amplification technologies (NAAT) to identify common pathogens have become a routine approach for infectious disease diagnosis in high-income countries. Many NAAT products now exist that have been cleared or approved by national health authority agencies, in addition to a variety of qualified laboratory-developed methods used in some facilities [2,3]. Although various amplification methodologies have been developed, RT-PCR is the predominant NAAT used for diagnostic testing [4]. The implementation of NAAT in clinical diagnostic laboratories is complex and can require sophisticated equipment, infrastructure, skilled laboratorians, and a robust quality management program [5]. These requirements present insurmountable challenges when trying to diagnose infectious diseases in the resource limited settings (RLS) of low and middle-income countries (LMIC), especially in rural environments where operational and logistical limitations further challenge implementation [4,6]. Patients with communicable diseases in these locations would greatly benefit from rapid on-site diagnosis and timely linkage to care, resulting in improved individual outcomes and better infectious disease control.

The multifaceted challenges facing NAAT point-of-care testing in LMICs demand solutions that can reliably integrate the often challenging nucleic acid (NA) extraction procedure, together with a suitable NA amplification/detection method. This unified system must be simple to operate, environmentally robust, yield timely results, be affordable and have high diagnostic accuracy. NAAT-based diagnostic platforms that are developed for use in high-income countries typically cannot tolerate the environmental extremes (e.g. temperature, humidity and dust) encountered in RLS and are not designed for use by healthcare workers that have not received extensive training [7,8]. These factors make most currently available sample-to-answer NAAT options unsuitable for implementation in peripheral facilities in LMICs [5,9]. A rapid time to result is necessary to reduce loss to follow up wherein the client may have multiple constraints that prevent returning for test results and possible treatment; these include distances required for travel to the test site, time-off from work for the visit, limited financial resources and other competing needs [10,11]. Currently, most NAAT test results for infectious diseases are not available on the same day [12–14]. Cost per test is a further barrier to adoption, as many LMICs cannot purchase these high-cost diagnostic tests without global donor support [15]. For example, the WHO published a target product profile for a simple and accurate diagnostic NAAT for *Mycobacterium tuberculosis* (MTB) to supplant smear microscopy with a targeted price of US $4–6 [16]. For such settings, there is a global need for innovators to design and develop low cost, fully integrated testing systems that meet required performance criteria, while being functional in austere environments and applicable for a variety of pathogenic agents.

As high quality NA extraction is a necessary first step toward an integrated platform, we assessed the performance of six NA extraction technologies to efficiently extract high quality NA from complex clinical sample matrices, such as sputum, stool and whole blood. A variety of metrics were used to evaluate and assess the performance of these six platforms, with the intent to determine if any might subsequently be integrated together with low-cost NA detection technologies, also suitable for RLS [4]. Blinded panels of quality controlled contrived specimens of sputum, blood, and stool, spiked with varying levels of bacterial and/or viral agents were used in a head-to-head challenge of these six NA extraction technologies. The performance of each was then compared using pathogen-specific real-time PCR assays for each target. Other factors important for RLS were assessed, including specimen input volume (largely for sensitivity), degree of automation, and processing time. Collectively, these data were compiled and analyzed to gauge the suitability of these six technologies for future inclusion into an LMIC-appropriate integrated testing system–a subject of ongoing interest to a funder, the Bill and Melinda Gates Foundation (BMGF).

## Materials and methods

### Identification of technology developers

An extensive search of peer-reviewed literature, published technical reports and white papers, commercial literature, company websites, conference proceedings, and letters of interest to a donor (BMGF) was conducted to identify multiple technology developers with innovative NA extraction methods with the potential for use in austere settings. Six developers agreed to participate in this study based upon their estimated potential to meet a critical cost goal of approximately $2 per extraction. The institutions included Akonni Biosystems, Inc. (Frederick, MD, USA), Claremont BioSolutions (Upland, CA, USA), Integrated Nano-technologies (Henrietta, NY, USA), MolBio Diagnostics (Bangalore, India), Paratus Diagnostics, (San Marcos, TX, USA) and the Hasleton group, Vanderbilt University (Nashville, TN, USA). Although these institutions had no role in designing the study, the authors agreed with the request that for purposes of anonymity, the identities of each developer and associated technologies would be coded for this report. These developers offered several innovative devices and NA extraction methods incorporating various chemical, heat, and/or mechanical lysis, with solid phase NA capture on silica or other NA-specific binding matrices coated onto beads, frits or other supports. The operating platforms ranged from prototype systems in development to further simplify workflow, to fully integrated devices. Each developer received identical blinded specimen panels to process in their facility. The isolated NAs were shipped to the Centers for Disease Control and Prevention (CDC) for subsequent molecular analysis.

### Specimen matrices

Multiple studies on the performance of NA platforms have been described previously thus this work utilized real time PCR analyses of sample extracts to establish if sufficient amounts and purity of target NAs were generated to give a positive result [17–19]. Human raw sputum, whole blood and stool specimen matrices were used in this study because they are common specimen types used for the diagnosis of many infectious diseases and because each is considered complex because of physiochemical attributes including viscosity, heterogeneity, extraneous NAs, commensal microflora, cellular debris and the presence of PCR inhibitors [20–26]. As the intent of this study was to directly compare different NA extraction methods, the use of contrived samples was deliberately chosen to limit the heterogeneity of each sample type as compared to clinically derived samples. In addition, the acquisition of sufficient amounts of excess or discarded clinical samples for use with multiple methods would have been extremely challenging. Other groups have described use of contrived samples in order to compare the performance of different extraction methods, test platforms and diagnostic laboratories hosting test platforms [19,27]. Aliquots of pooled raw human sputum (10 X 25 mL volumes) and stool (3 X 75 g) were procured from BioIVT (Westbury, NY, USA). Bloodworks Northwest (Seattle, WA, USA) provided one unit (~ 400 mLs) of human whole blood. Total NAs were first extracted from these specimens and screened via real-time RT-PCR at PATH using the protocols described below to confirm the absence of the target microbes included in in the primary specimens. The King County Tuberculosis Laboratory (Seattle, WA, USA) screened aliquots from the pooled sputum samples for the presence or absence of MTB via mycobacterial growth indicator tubes (MGIT, Becton Dickinson, Franklin Lakes, NJ, USA) and Löwenstein–Jensen and Middlebrook 7H10 agar slants.

### Culture and preparation of microbial agents for specimen spiking

Each specimen type (stool, sputum, or whole blood) was spiked with bacteria and/or an RNA-based virus to enable semi-quantitative PCR assessment of both DNA and RNA extraction efficiencies from each specimen panel. All of the real time PCR assays used in this study are qualitative and not quantitative. To assess extraction performance, the specimen matrices were spiked with target microbes at levels that represented high, medium and low levels of target. It was assumed that all methods assessed in this exercise would detect the high levels and likely the medium range with the detection of low levels from extracts being the primary indicator of performance in terms of recovery and purity of the extract. MTB H37Rv strain Johannesburg and a clinical isolate of influenza A (H3N1) were used for sputum spiking; *Salmonella enterica* serovar Typhimurium LT2 and male specific 2 (MS2) bacteriophage were used for stool; and *Streptococcus pneumoniae* and MS2 phage were used for whole blood. The inactivated MTB cells were procured from the laboratory of Dr. Bavesh Kana (University of Witwatersrand, Johannesburg, South Africa) and supplied in ten mLs of phosphate buffered saline (PBS) [28]. The MTB cells were originally cultured in Middlebrook 7H9 broth supplemented with 5% glycerol with incubation at 37°C. The MTB cells were chemically inactivated to ensure minimal risk from laboratory-acquired infection during processing. No growth in MGIT after 42 days incubation confirmed the inactivation of MTB. The influenza A strain was an H3N1 clinical isolate provided by the Washington State Department of Health Public Health laboratories (Shoreline, WA, USA). Dr. Scott Meschke (University of Washington, Seattle, WA, USA) gifted both MS2 phage and *S*. Typhimurium LT2. MS2 phage was propagated in an *Escherichia coli* F^amp+^ strain as previously described [29], harvested from the soft agar overlay, filtered through a 0.22 μm filter (EMD Millipore, Billerica, MA, USA), and stocks stored at 4°C until use. *S*. Typhimurium LT2 was cultured in tryptic soy broth (Sigma-Aldrich, St Louis, MI, USA) with overnight incubation at 37°C and shaking. The cells were pelleted by centrifugation, washed once with PBS, resuspended in 20 mLs PBS, and stored at 4°C until use. *S*. *pneumoniae* (American Type Culture Collection, Manassas, VA, USA) was cultured at 37°C overnight in brain-heart infusion broth (Sigma-Aldrich). The cells were pelleted by centrifugation, washed once with PBS and resuspended in RPMI-1640/15% glycerol (Sigma-Aldrich) to prevent autolysis [30]. The cell suspension was aliquoted in 1mL stock volumes and stored at ‒80°C until use.

### Reference nucleic acid extraction methods for panel construction

With the exception of MTB, the extraction of the bacterial and viral NAs at the PATH laboratory was performed using Qiagen NA extraction kits (Hilden, Germany) as described below. All NA eluates derived from extractions were stored at -80°C until use.

Viral RNA from spiked sputum and blood panel members was extracted using 140 μL aliquots of each specimen, processed with the QIAamp viral RNA mini kit following the manufacturer’s protocol. All RNAs were eluted in a final volume of 60 μL. For viral RNA from spiked stool panel members, a pre-RNA extraction clean-up step was done as follows. Briefly, 200 mg amounts of stool were homogenized in 800 μL PBS via vortexing and then clarified by centrifugation for 20 minutes at 4,000 X *g*. The supernatant was filtered using a 0.22 μm filter (EMD Millipore), and 140 μL aliquots of filtrate were then processed with the QIAamp viral RNA mini kit. Bacterial DNA from blood was extracted using 200 μL processed with the QIAamp DNA blood mini kit and eluted in a final volume of 200 μL. Bacterial DNA from stool was extracted from 200 mg using the QIAamp DNA Stool mini kit and eluted in 200 μL volumes. MTB DNA was extracted from sputum using a previous methodology [31]. Briefly, 500 μL sample volumes were liquefied using an equal volume of 2% N-acetyl-L-cysteine and incubated at room temperature for 15 minutes. PBS was added to a final volume of 50 mLs, the cells pelleted by centrifugation, resuspended in 100 μL Tris EDTA buffer (pH8.0) containing acid-washed glass beads and disrupted for 2 min (Cell Disruptor Genie, Scientific Industries, Bohemia, NY, USA). The cell lysates were centrifuged and clarified supernatants transferred to sterile microtubes.

Spiked target levels (described below) for the microbes in each panel were independently confirmed at the CDC laboratory as follows. Briefly, all spiked samples were extracted using the MagNA Pure Compact instrument with the Total Nucleic Acid Isolation Kit I external lysis protocol (Roche Applied Science, Indianapolis, IN, USA) and eluted in a final volume of 100 μL for all extracts. Prior to NA extraction, the different specimen types were processed via alternative methods. For sputum, 300 μL of each sample was first incubated with 300 μL dithiothreitol solution (ThermoFisher Scientific, Waltham, MA, USA) at room temperature for 1 hour prior to extraction. For blood samples, 400 μL of blood was first mixed with 400 μL bacterial lysis buffer (Roche), 400 μL pH neutral buffer, and 40 μL proteinase K (25 mg/mL) with subsequent incubation at ambient temperature for 15 minutes. Glass beads (800 mg, 0.5 mm) were added and the cells were disrupted twice for one minute at 5000 rpm. The solution was then centrifuged at 10 000 × *g* for one minute and 700 μL of the supernatant used for extraction. For stool, 200 mg were mixed with 2 mL PBS and then briefly centrifuged to pellet particulates. Four hundred μL of the supernatant was combined with 400 μL bacterial lysis buffer (Roche), 400 μL pH neutral buffer, and 40 μL proteinase K (25 mg/mL) and incubated at ambient temperature for 15 minutes. Glass beads (800 mg, 0.5mm) were added and cells disrupted twice for one minute at 5000 rpm. The solution was centrifuged at 10 000 × *g* for one minute, and 700 μL of supernatant was used for extraction. All NA eluates were stored at -80°C until use.

### Reference methods for RT-PCR detection of nucleic acid targets for panel construction

The PATH and the CDC laboratories both used the CDC-validated real-time RT-PCR assays specific for each target microbe in this study. Oligonucleotide primers and probes were procured from IDT (Coralville, IA, USA). Real time PCR has previously been described as an effective method by which to assess the performance of extraction methods in that it is highly specific, it can detect very low levels of target and can indirectly inform on the purity of extracted samples [17–19]. The core amplification reagent used in all real time RT-PCR assays was qScript XLT 1-Step RT-qPCR ToughMix (Quanta Bio, Gaithersburg, MD, USA). All reactions were conducted in 20 μL volumes into which 5 μL of NA extract was added to create a final reaction volume of 25 μL. All reactions were processed under the same thermal cycling protocol, irrespective of the RT-PCR instrument used or the target microbe and the specimen matrix, and included use of a reverse transcription step. Cycling conditions were as follows: 45°C for 10 min, 95°C for 10 min, 45 cycles of 95°C for 30 sec and 60°C for 1 min. Fluorescence in the FAM channel was measured in real time during the 60°C stage. Real-time RT-PCR was performed on an Agilent Mx3005P qPCR system (Agilent Technologies, Santa Clara, CA, USA) at the PATH laboratory and the Applied Biosystems 7500 Fast system (ThermoFisher Scientific, Waltham, MA) at the CDC laboratory.

#### Construction of DNA- and RNA-spiked blinded panels

Each pooled specimen panel was comprised of ten sample members, with most spiked with a bacterial pathogen and/or an RNA-based virus to assess NA extraction efficiencies from the three specimen matrices (Table 1). Each panel was comprised of seven spiked (positive) and three unspiked (negative) samples. These were labelled SP1–10 (SPutum), BL1–10 (BLood), and ST1–10 (STool). Sputum was spiked with MTB and/or influenza A virus; whole blood was spiked with *S*. *pneumoniae* and/or MS2 bacteriophage; and stool was spiked with *S*. Typhimurium LT2 and/or MS2 phage. The input amount of each test microbe used for spiking was semi-quantitatively determined via PCR Crossing threshold (C_t_) values derived from the NA extracts of the various agents. The intent was to spike samples with sufficient NA to represent high (C_t_ 18 ± 1.5), medium (C_t_ 26 ± 1.5) and low (C_t_ 35 ± 1.5) amounts of test microbe NA in each specimen panel, with corresponding levels of one microbe and/or the other [19].

**Table 1:** Target-spike levels used in the construction of each specimen panel. The format of each panel was the same. Samples were spiked with different microbes to represent varying levels of DNA and RNA for subsequent NA extraction: +++ high spike (C_t_ 18 ± 1.5); ++ medium spike (C_t_ 26 ± 1.5); + low spike (C_t_ 35 ± 1.5);–no spike.

Sample	RNA-based microbe	DNA-based microbe
**1**	+	+++
**2**	-	-
**3**	-	+++
**4**	+++	-
**5**	-	+
**6**	-	-
**7**	+++	+
**8**	-	-
**9**	-	++
**10**	++	-

The sputum (SP) panel was prepared by pooling 210 mLs of raw human sputum and blending to create a homogenous mixture. The homogenate was then split into ten 20 mL aliquots. Eight aliquots were spiked with MTB cells and/or influenza A virions, again corresponding to high, medium, and low C_t_ levels, and subsequently mixed to create a uniform distribution of MTB cells and influenza A virions in the respective test panel samples. The SP panel samples were dispensed into 0.5 mL aliquots for later use.

Whole blood (BL) was split into ten 36 mL aliquots. Eight aliquots were spiked with varying concentrations of *S*. *pneumoniae* and MS2 phage corresponding to high, medium, or low C_t_ levels. The spiked blood panels were then mixed to ensure an even distribution of spiking agents. The ten BL panels were then aliquoted in 1 mL volumes.

For the construction of the stool (ST) panel, a 40% (w/v) stool homogenate in PBS was prepared in a blender and aliquoted into ten 40 mL volumes. Eight samples were spiked with *S*. Typhimurium and/or MS2 stocks corresponding to high, medium, or low C_t_ levels and distributed into 1 mL aliquots. After each sample set was prepared, the tubes were frozen using a dry ice/ethanol bath and then stored at -80°C until shipping or further use.

To ensure the C_t_ value from each spiked sample was within the expected range, the DNA and RNA were extracted from representative aliquots in each panel following their construction. The NA extracts were then analyzed by real time RT-PCR assays specific to each spiked microbe. Low-spiked samples representing each target microbe were also processed after a freeze and thaw cycle to ensure sample integrity. NA extracts from the three specimen panels were then independently tested by RT-PCR at the CDC to confirm the original test data derived at the PATH laboratory. While the C_t_ values were typically in close agreement to the PATH NA extracts, trace amounts of MTB DNA were consistently detected in Sample SP6, a supposedly MTB negative sample. This sample was removed from the sputum panel and therefore the final SP panel was comprised of only nine samples, each of which were present in triplicate aliquots. The 10 panel members for the remaining blood and stool panels were each presented in triplicate.

The 87 test aliquots (less the three replicates of SP6) across the three specimen panels were blinded via four-digit numbers and shipped overnight on dry ice to the developers with confirmation of the integrity of cold chain upon receipt. Each developer was asked to document the amount of each sample used for each NA extraction, the final elution volume, the time required and the number of user steps for extraction each panel. All NA extracts from each developer were frozen and then shipped to the CDC laboratory on dry ice via overnight courier. The extracts were stored at -80°C until testing. Ultimately, each blinded NA extract was tested in triplicate via the CDC real time RT-PCR protocols for the corresponding microbial NAs (three PCR reactions for each triplicate aliquot per panel member), and the C_t_ values shared with the PATH laboratory for subsequent analyses.

#### The use of polar plots to rank technologies on multiple key attributes

The WHO created the acronym ASSURED to list the key attributes necessary for an effective point of care test, e.g. a rapid diagnostic test [32]. We can apply many of these attributes to the NA extraction technologies screened in this study except for E (Equipment-free) and D (Deliverable) since all of the technologies require a device and none of these technologies are currently manufactured or marketed at scale. In terms of A (Affordable), all of the developers consider their technology costs $2 per test and so all were scored at this price. The SS (Sensitive and Specific) data points are addressed with the analytical performance data described in the results section. To address U (User-friendly) and R (Rapid), we applied the number of steps required to perform each extraction and the turnaround times for each method, respectively. Three polar plots, one for each specimen panel, were generated for each developer based on the two target NAs within each panel. All of the ASSUR components were individually normalized in a range from low (0.0) to high (1.0) scores for each metric with 1.0 being the most optimal value measured in each category. The five normalized parameters of ASSUR calculated each plot were equally weighted and the average of these parameters were calculated for each target microbe. These averages were then pooled and re-averaged based on the sample type and also for all three sample types and these values used to rank developers in terms of ASSUR.

## Results

### Real time RT-PCR qualification of microbe-spiked challenge panels

The real time RT-PCR C_t_ values from the original microbe-spiked challenge panels tested by PATH (predominantly by Qiagen kits) were used as the reference standard to compare the various NA extracts from sputum, blood and stool (see PATH columns, Table 2). The CDC further qualified the three panels by ensuring that each specimen yielded similar results to the PATH analyses after freezing, shipping, nucleic acid extraction via a different platform, the Roche MagNaPure and analysis by real-time RT-PCR analysis (see CDC columns, Table 2). The data from the SP and BL panels extracted by the MagNaPure (CDC) closely correlated with the Qiagen (PATH) data with a +/- 2 variance observed between the mean C_t_ values (Table 2). However, the stool panel data showed greater variance. Although the MS2 C_t_ values were similar between the CDC and PATH results (Table 2), less *S*. Typhimurium DNA was detected in the CDC NA extracts from the mid- and high-spiked ST panel members, with no amplification detected in the low-spiked samples. This may be due to PATH using two separate Qiagen kits to extract DNA and RNA respectively, from stool in order to optimize NA recoveries. The CDC laboratory performed a single total NA extraction method with the MagNaPure that incorporated a brief centrifugation of liquefied stool samples to reduce debris prior to extraction. This centrifugation step may explain the differences seen in *S*. Typhimurium DNA levels, as many of the bacterial cells would likely have been removed in this step. With the exception of *S*. Typhimurium, the CDC results verified the target NAs levels in all panels (Table 1) and indicated that both the Qiagen and MagNaPure methods used in either laboratory gave typically similar RT-PCR results. All annotated and complete real time RT-PCR data sets from each group is available online (https://dataverse.harvard.edu/dataverse/SSB).

**Table 2:** Mean C_t_ values derived from real time RT-PCR analyses of nucleic acid extracts prepared by different extraction methods using the blood, stool and sputum test panels. Shaded and non-shaded rows represent spiked (positive) and non-spiked (negative) samples, respectively; A–F represent coded identifiers of the six developers; the CDC used the Roche MagNaPure platform; PATH used the Qiagen extraction kits except for MTB; SP, sputum; BL, blood; ST, stool; ND no amplification detected; ^a^ 1–2 false negative RT-PCR replicate(s) detected within a confirmed positive member; ^b^ false positive RT-PCR replicate(s) detected within negative sample; Sens, mean sensitivity across all nine replicates; Spec, mean specificity across all nine replicates; CI, confidence interval. Dark shading indicates spiked samples; NC, not calculated.

	Developer								Developer							
	A	B	C	D	E	F	CDC	PATH	A	B	C	D	E	F	CDC	PATH
Panel	Influenza A								*M*. *tuberculosis*							
SP1	ND	ND	37.4	ND	32.6 ^a^	ND	32.2	33.9	15.2	28.5	17.6	33.6 ^a^	20.9	16.7	16.9	16.8
SP2	ND	ND	ND	ND	ND	ND	ND	ND	ND	ND	ND	36.5 ^b^	ND	ND	ND	ND
SP3	ND	ND	ND	ND	24.8 ^b^	ND	ND	ND	15.2	29.9	17.5	34.9 ^a^	21.9	16.7	16.6	17.1
SP4	22.3	ND	21.8	ND	23.3	24.8	21.2	19.6	37.5 ^b^	ND	ND	ND	ND	ND	ND	ND
SP5	ND	ND	ND	ND	ND	ND	ND	ND	34	ND	36.1 ^a^	ND	37.9 ^a^	35.3 ^a^	36.1	33.9
SP7	22	ND	26.4	ND	23.5	25	21.2	19.6	34	ND	36.2 ^a^	ND	41.3 ^a^	ND	34.9	35.9
SP8	ND	ND	ND	ND	ND	ND	ND	ND	ND	ND	ND	ND	ND	ND	ND	ND
SP9	ND	ND	ND	ND	ND	ND	ND	ND	22.9	ND	26.1	ND	32.1	25.0	25.9	26.4
SP10	29.1	ND	28.8	ND	30.3	31.9	28.2	26.5	ND	ND	ND	ND	ND	ND	ND	ND
Sens 95% CI	0.75 0.58,0.88	0.00 0.00,0.10	1.00 0.90,1.00	0.00 0.00,0.10	0.86 0.71,0.95	0.75 0.58,0.88	NC	NC	1.00 0.92,1.00	0.40 0.26,0.56	0.86 0.71,0.95	0.33 0.20,0.49	0.76 0.60,0.87	0.71 0.56,0.84	NC	NC
Spec 95% CI	1.00 0.92,1.00	1.00 0.92,1.00	1.00 0.92,1.00	1.00 0.92,1.00	0.93 0.82,0.99	1.00 0.92,1.00	NC	NC	0.86 0.71,0.95	1.00 0.90,1.00	1.00 0.90,1.00	0.92 0.78,0.98	1.00 0.90,1.00	1.00 0.90,1.00	NC	NC
Rank	3	5	1	6	2	3			1	5	1	6	3	4		
Panel	MS2 bacteriophage								*S*. *pneumoniae*							
BL1	31.0	32.4 ^a^	ND	ND	36.5 ^a^	32.8	33.4	32.3	20.6	27.6	25	30.5	23.9	21.9	19.2	20.9
BL2	ND	34.9 ^b^	ND	ND	ND	ND	ND	ND	ND	ND	ND	ND	ND	ND	ND	ND
BL3	ND	34.8 ^b^	ND	ND	ND	ND	ND	ND	20.8	27.8	24.7	29.7	24.2	22.5	19.1	20.8
BL4	17.5	23.6	34	ND	23.2	18.8	19.4	19.7	ND	ND	ND	ND	ND	ND	ND	ND
BL5	ND	36.4 ^b^	ND	ND	ND	ND	ND	ND	32.1	ND	37.1 ^a^	37.0 ^a^	35.8	37.0 ^a^	31.9	33.3
BL6	31.1	35.6	ND	ND	36.5 ^a^	32.7	33.3	33.5	ND	ND	ND	ND	ND	ND	ND	ND
BL7	17.0	23.8	33.9 ^a^	38.1 ^a^	22.5	19.4	19.6	20.7	34.5	ND	35.7 ^a^	37. ^a^	36.6 ^a^	37.0	32	33.0
BL8	ND	36.1 ^b^	ND	ND	ND	ND	ND	ND	ND	ND	ND	ND	ND	40.4 ^b^	ND	ND
BL9	ND	36.2 ^b^	ND	ND	ND	37.6 ^b^	ND	ND	28	34.1	30.3	36.6 ^a^	29.9	30.8	26.1	28.0
BL10	24.0	30.9	ND	ND	29.8	26.7	26.5	24.9	ND	ND	ND	ND	ND	39.3 ^b^	ND	ND
Sens 95% CI	1.00 0.92,1.00	0.93 0.82,0.99	0.36 0.22,0.51	0.07 0.01,0.18	0.87 0.73,0.95	1.00 0.92,1.00	NC	NC	1.00 0.92,1.00	0.60 0.44,0.74	0.73 0.58,0.85	0.60 0.44,0.74	0.96 0.85,0.99	0.98 0.88,1.00	NC	NC
Spec 95% CI	1.00 0.92,1.00	0.02 0.00,0.12	1.00 0.92,1.00	1.00 0.92,1.00	1.00 0.92,1.00	0.98 0.88,1.00	NC	NC	1.00 0.92,1.00	1.00 0.92,1.00	1.00 0.92,1.00	1.00 0.92,1.00	1.00 0.85,0.99	0.91 0.79,0.98	NC	NC
Rank	1	6	4	5	3	2			1	5	4	5	2	3		
Panel	MS2 bacteriophage								*S*. Typhimurium							
ST1	38.1 ^a^	ND	38.6 ^a^	ND	ND	41.6 ^a^	36	34.4	24.5	28.7	35.3	32.1 ^a^	26	25.5	26.4	22.7
ST2	ND	ND	ND	ND	ND	ND	ND	ND	ND	ND	ND	ND	ND	ND	ND	ND
ST3	ND	ND	ND	ND	ND	ND	ND	ND	21	29.6	30.1	29.3 ^a^	25	26.6	26.5	23.2
ST4	21.1	23	24.3	27.5	23.9	22.1	22.6	16.3	ND	ND	ND	ND	ND	ND	ND	ND
ST5	ND	38.0 ^a^	ND	ND	ND	ND	ND	ND	30.7	ND	ND	39.0 ^a^	ND	ND	ND	32.9
ST6	36.8 ^a^	ND	37.8	37.9 ^a^	ND	42.4 ^a^	36	32.1	ND	ND	ND	ND	ND	ND	ND	ND
ST7	20.9	23.8	23.9	27.9	25.5	21.8	22.8	15.9	30.4	ND	ND	37.3 ^a^	ND	ND	ND	33.0
ST8	ND	31.2 ^b^	38.4	ND	ND	ND	ND	ND	ND	ND	ND	ND	ND	ND	ND	ND
ST9	ND	35.5 ^b^	ND	ND	ND	ND	ND	ND	25.6	31.3	ND	32.4	29.0	30.9	31.6	27.9
ST10	29.3	31.1	32.9	38.7 ^a^	35.1	30.8	32.9	24.2	ND	ND	ND	ND	ND	ND	ND	ND
Sens 95% CI	0.84 0.71,0.94	0.60 0.44,0.74	0.73 0.58,0.85	0.60 0.44,0.74	0.67 0.50,0.80	0.80 0.65,0.90	NC	NC	1.00 0.92,1.00	0.60 0.44,0.74	0.40 0.26,0.56	0.64 0.49,0.78	0.57 0.41,0.72	0.60 0.44,0.70	NC	NC
Spec 95% CI	1.00 0.92,1.00	0.53 0.38,0.68	1.00 0.92,1.00	1.00 0.92,1.00	1.00 0.92,1.00	1.00 0.92,1.00	NC	NC	1.00 0.92,1.00	1.00 0.92,1.00	1.00 0.92,1.00	1.00 0.92,1.00	1.00 0.92,1.00	1.00 0.92,1.00	NC	NC
Rank	1	6	3	5	4	2			1	3	6	2	5	3		

### Analytical sensitivity and specificity of the developer NA extracts from the challenge panels

All developers completed the NA extractions using their respective technologies and shipped the extracts to the CDC. Two minor deviations were observed: Developer E shipped only two of the three ST3 and ST6 samples provided to them; and one extract of sample SP3 from developer C contained insufficient elution volume only allowing for influenza A screening and not MTB.

With the above exceptions, NA extracts derived from all triplicate panel members were tested in triplicate by real-time RT-PCR, resulting in nine data points per panel member. The mean C_t_ values were calculated for the microbe NAs in each panel sample (Table 2). Sensitivity and specificity calculations for each developer’s technology were scored against the CDC and PATH reference datasets, along with their 95% confidence intervals (summarized in Table 2). Some NA extracts were retested after initial screening to confirm whether procedural errors accounted for unexpected results. This root cause analysis enabled the identification of mislabeled samples from two developers, and their final RT-PCR data were corrected based on repeat testing to account for the original labelling errors. Complete and annotated data sets for each developer are publicly available (https://dataverse.harvard.edu/dataverse/SSB).

### Sputum panel results

Developer C extracted influenza A RNA from all spiked samples with sensitivity of 100%. Developer E extracted most influenza A RNAs, but not all RT-PCR tests were positive when using low sample extracts (e.g. SP1), with a further three false positive replicates reported from one extract (SP3) indicating potential cross contamination. Analyses of the Influenza A RNA produced by Developer E’s extraction technology showed an overall sensitivity of 86% for Influenza A RNA. Influenza A RNA from the medium (SP10) and high (SP4 and SP7) spiked samples was produced at detectable levels by two developers (A and F), but the low-level sample (SP1) was scored as negative with these technologies. Developers B and D did not produce Influenza A RNA in any SP extracts, even those with high-spike levels. When specificity was considered, most developers scored 100%, with the exception of developer E scoring 93%. Overall, developer C had the highest concordance with the predicate methods for influenza A RNA extraction from sputum, namely Qiagen manual extraction (PATH) or the MagNaPure (CDC).

Better overall extraction performance was observed with MTB (Table 2). Developer A correctly extracted MTB DNA from all spiked samples, whereas developers C and E managed to produce MTB DNA from most MTB spiked samples, though not as consistently from three replicates from the MTB low-level spiked samples (SP5 and SP7). Developers B and D were only able to extract detectable MTB DNA from the samples with the highest-spiked levels (SP1 and SP3). Of note are the MTB DNA extracts from developer A where the C_t_ values generated from testing were generally lower than the MagNaPure (CDC) and manual (PATH) data sets (Table 2), indicating that for MTB, the developer A extraction method was more efficient than the PATH or CDC reference methods. Analyses of the extracts from developer A achieved a sensitivity of 100%, whereas extracts from developers B, D, E and F demonstrated significantly lower sensitivities. The extracts from most technologies scored well with regards to specificity, with developers B, C, E and F achieving 100%, however developer A scored least well of the six groups with their extracts generating a specificity of 86%, possibly due to cross contamination affecting the MTB-negative sample, SP4 (Table 2). Overall, for MTB DNA extraction in sputum, developer A had concordance to the reference methods amongst the six technologies, but developer C also scored well. For the sputum panel overall, developer C demonstrated the highest concordance to the reference methods when considering the simultaneous extraction of both DNA and RNA targets.

### Blood panel results

The extracts from developers A, B, E and F all contained detectable amounts of MS2 RNA across the spiking range from high to low (Tables 1 and 2). However, only extracts from developer A had 100% agreement for all replicates compared to the MagNaPure (CDC) and Qiagen (PATH) reference data. With MS2, developer A extracts gave consistently lower C_t_ values than the reference data indicating the most effective purification overall. Extracts from developer F correctly corresponded to the positive samples, but an extract from a negative sample, BL9, gave a false positive RT-PCR result, presumably because of a cross contamination event. Developer E detected some low-spiked sample replicates (BL1 and BL6) but correctly detected all replicates from the higher-spiked panel members (BL4, BL7, and BL10). RT-PCR on the extracts from developer B correctly identified the majority of replicates spiked with MS2, but surprisingly, many of the unspiked sample extracts also scored RT-PCR positive (Table 2), resulting in poor specificity for this panel. Upon investigation it was discovered that developer B routinely used MS2 in other development work in their laboratory, so it is possible that reagents were contaminated with MS2 phage or MS2-related amplicons. Samples from developers C and D were non-reactive for 3/6 and 4/6, respectively, of the 6 MS2-spiked samples, resulting in poor sensitivity. The observed specificity for developers A, C, D and E was 100% with developer F closely following at 98%. Overall, developer A ranked highest with the MS2 challenge in whole blood with 100% sensitivity and specificity. Developer F followed with an observed sensitivity and specificity of 100% and 98%, respectively. Developer E has slightly lower sensitivity of 87% with a specificity of 100%.

The MagNaPure extracts gave the lowest C_t_ values across the panel range with *S*. *pneumoniae* DNA extraction from whole blood. Many of the developer’s technologies demonstrated good performance with all spiking ranges correctly generating RT-PCR positive results (Table 2). However, only the extracts from developer A were consistently RT-PCR positive with all spiked replicates, resulting in a sensitivity of 100%. The replicate extracts from the low-spiked samples (BL5 and/or BL7) as prepared by developers C, D, E, and F generated variable RT-PCR results with developers E and F showing higher sensitivities at 96% and 98%, respectively. All technologies achieved ≥90% specificity, with developers A, B, C, D and E scoring 100%. Factoring in both MS2 RNA and *S*. *pneumoniae* DNA extractions from whole blood, developer A scored best overall with similar C_t_ values to the reference assays used by the CDC and PATH.

### Stool panel results

The MS2 RNA extractions from stool panel were the only test panel where no developer achieved 100% sensitivity, as not all replicate extracts derived from the low-spiked samples (ST1 and ST6) gave positive RT-PCR results (Table 2). The MagNaPure assay also performed poorly as compared to the Qiagen method employed at PATH. The extracts from developers A and F scored highest sensitivity with 84% and 80%, respectively. In terms of specificity, developer B extracts again appeared to be confounded by potential MS2 contamination, but to a lesser degree than observed with the blood panel. The extracts from other developers demonstrated 100% specificity. Overall, developer A had the greatest concordance for extracting MS2 RNA as compared to the six technologies, with developer F being a close second. All of the positive samples had C_t_s ≥4.8 higher than the corresponding PATH data, indicating that the extraction and/or the co-purification of confounders had significant negative effects on generating a test result.

For *S*. Typhimurium, developer A was the only technology whose NA extracts were detected with 100% sensitivity. Interestingly, the sensitivities scored for extracts from the other developers were all relatively low compared to the other microbe panels, with developer D as the next best at 64%. All technologies scored 100% for specificity. With the stool panel, developer A had significant concordance with the data derived from the different extraction methods used at PATH and CDC than compared to any of competitors in terms of both RNA and DNA extraction followed by developer F.

### Volumetric effects on the performance of NA extraction

As with most NAATs, multiple factors may contribute to the diagnostic accuracies observed with these six technologies. These include the amount of sample processed in a NA extraction, the final elution volume, and the percentage of the eluate used in the amplification/detection steps. Theoretically, processing a large amount of specimen and/or eluting captured NAs in a smaller volume can result in a more concentrated sample, thus increasing the likelihood of amplification target NA. The specimen input and elution volumes of each NA extraction method varied substantially among the developers (Table 3). Inputs ranged from 80–500 μL for sputum processing, 50–1000 μL for blood, and 70–750 μL for stool. Elution volumes ranged from 50–105 μL and were consistent across specimen types within each technology. The percentage of eluate among the six technologies used in the RT-PCR step varied less, ranging between 4.8–10%.

**Table 3:** The comparative assessment of sample input amounts and NA elution volumes used by each developer. The amounts of samples provided in each panel is shown alongside the amount of sample used for NA extraction by each developer in addition to the elution volume. The percentage of NA eluates used for each RT-PCR reaction (5 μL per reaction) and the percentage of sample used per RT-PCR reaction are shown with the ranking of pooled sensitivity and specificity observed from each specimen type. NA; nucleic acid.

Group	Specimen type	NA extracted	Sample input (μL or mg stool)	% of total sample	Elution volume (μL)	% of eluate used for RT-PCR	% of sample used for RT-PCR
PATH	Sputum	DNA	500	100	100	5.0	5.0
		RNA	140	28	60	8.3	2.3
	Blood	DNA	200	20	200	2.5	0.5
		RNA	140	14	60	8.3	1.2
	Stool	DNA	200	20	200	2.5	0.5
		RNA	28	3	60	8.3	0.2
CDC	Sputum	DNA/RNA	300	60	100	5.0	3.0
	Blood	DNA/RNA	225.8	23	100	5.0	1.2
	Stool	DNA/RNA	22.6	2	100	5.0	0.1
A	Sputum	DNA/RNA	500	100	105	4.8	4.8
	Blood	DNA/RNA	1000	100	105	4.8	4.8
	Stool	DNA/RNA	750	75	105	4.8	3.6
B	Sputum	DNA/RNA	500	100	150	3.3	3.3
	Blood	DNA/RNA	100	10	150	3.3	0.3
	Stool	DNA/RNA	250	25	150	3.3	0.8
C	Sputum	DNA/RNA	150	30	50	10.0	3.0
	Blood	DNA/RNA	50	5	50	10.0	0.5
	Stool	DNA/RNA	70	7	50	10.0	0.7
D	Sputum	DNA/RNA	80	16	100	5.0	0.8
	Blood	DNA/RNA	80	8	100	5.0	0.4
	Stool	DNA/RNA	80	8	100	5.0	0.4
E	Sputum	DNA/RNA	100	20	50	10.0	2.0
	Blood	DNA/RNA	100	10	50	10.0	1.0
	Stool	DNA/RNA	77	8	50	10.0	0.8
F	Sputum	DNA/RNA	500	100	100	5.0	5.0
	Blood	DNA/RNA	250	25	100	5.0	1.3
	Stool	DNA/RNA	150	15	100	5.0	0.8

We examined the relationship between sample volume and elution volumes with respect to the volume of eluate used for RT-PCR and back calculated to the amount of sample per RT-PCR reaction. These were then compared to the overall performance ranking of each developer using the pooled sensitivity and specificity data for each panel (Table 2). Developer A, the overall top performer in terms of NA extraction methods being assessed, typically used the largest amount of input material (75% - 100% of available material) with NA elution in a final volume of 105 μL which resulted in a high percentage of sample being used. Developer F had the greater relative amount of sample to input (100%), but only with the MTB panel. Developers B and F also used larger sputum input and NA elution volumes, but did not achieve the level of sensitivity seen with developer A. Developer E, who ranked third with all matrices in terms of sensitivity, used smaller amounts of input sample (8–20% of available material) and eluted in the smallest volume recorded (50 μL). Thus, their extraction process was efficient in terms of extraction methodology. The developers C and D used small sample inputs (7%- 30%) and also had poorer performance based on sensitivity, with the exception of sputum for which developer C scored best overall using only 16% of the sputum sample. Developer B used small sample inputs for blood and stool (10% and 25% respectively) and eluted in the largest eluate (150 μL) noted amongst the six groups; this combination may have contributed to the typically lower sensitivity observed across all panels (Table 2).

### Workflow assessment—Operator steps and processing times of candidate extraction technologies

**Fig 1:**
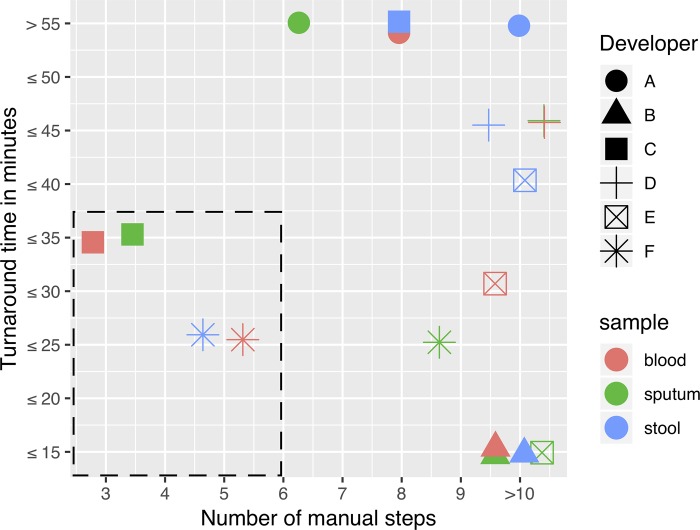
A comparison of sample processing times and manual steps to extract NAs from sputum, blood and stool specimens. Comparison or turnaround time (min) and number of manual steps for extraction methods for developers (A-F) for each specimen type (sputum, blood, and stool). The area surrounded by the dashed line indicates where the more optimal technologies are in terms of processing time and the number of user steps. Note, to prevent obfuscation with clusters of multiple symbols located on a single point, in such instances each symbol is slightly diffused (where applicable) to provide clarity to the reader.

The ideal workflow for NAAT in RLS should be rapid and require minimal hands-on processing. Each developer documented the turnaround time to process each specimen type, as well as the number of manual operator steps required for the workflow (Fig 1). All methods required less than one hour to complete, however processing time differences ranged from ≤ 15 min to ≥ 55 min, with developers B, E and F requiring the shortest turnaround times. Stool samples required longer processing times for developers C and E (30 and 20 minutes respectively) as compared to sputum or blood, due to additional steps to liquefy and clarify stool before the NA extraction step (Fig 1). This was also evident with the work stream for developer C who required only three operator steps for sputum and blood, yet eight for stool. Protocols from four developers included ten operator steps for stool sample processing, reflecting that these technologies are relatively early in workflow development or are less integrated compared to others. The protocol with the shortest turnaround time (developer B) had the most hands-on steps and the poorest overall performance. Developer F had a consistently short turnaround time with ≤ 25 minutes for all matrices. Ideally, the optimal technology will have a minimal numbers of operator steps prior to fully automated NA extraction. Developer A, the best overall performer, had the longest turnaround time (≥55 minutes) and 6 - ≥10 manual steps depending on the sample type processed. Developer C had the fewest number manual steps and processing times (< 35 minutes) for sputum and blood; stool processing required 1 hour.

#### Ranking of developers based on key attributes

**Fig 2:**
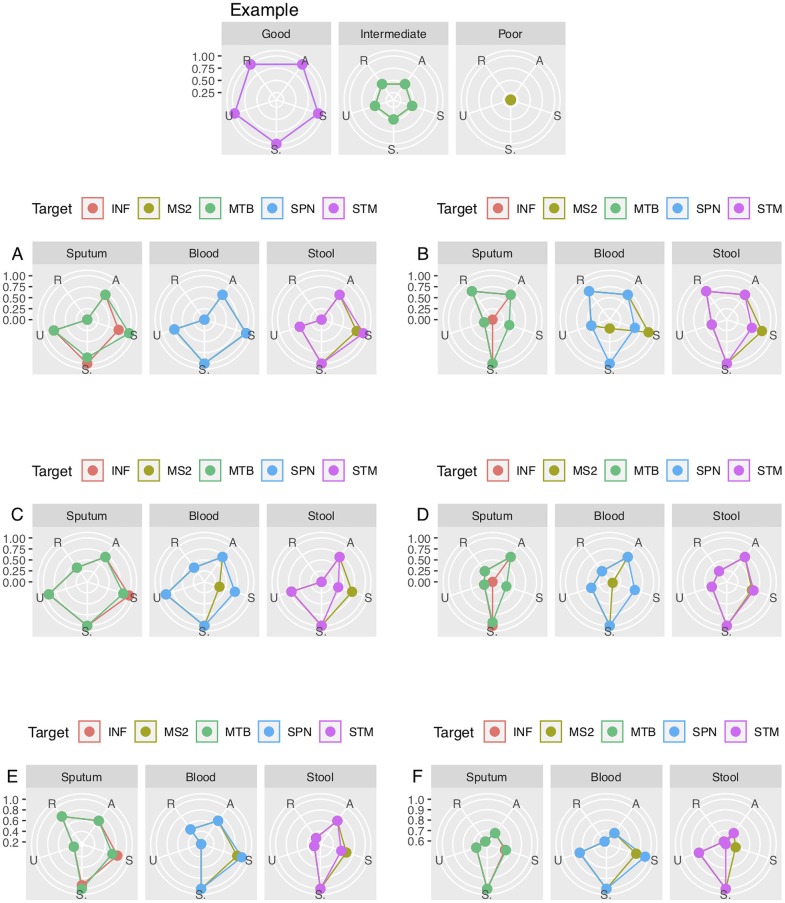
**The comparison of each technology (developer A–F) via radar plots using ASSUR.** The ASSUR criteria, as defined in the text, was applied for each target microbe used in the three specimen panels of sputum, blood and stool. Each component for ASSUR was normalized from 0.0 to 1.0 where 1.0 represents the most optimal value within each component in the set of data points for each component. The uppermost set of three images are examples to indicate the polar curves generated to indicate analysis via ASSUR as Good (1.0), Intermediate (0.5) and Poor (0.0) respectively. The developers are listed as A–F and with panels for sputum (SP), Blood (BL) and sputum (SP) and plots for each target nucleic acid associated within each panel. When the radar plot is read clockwise the letters read ASSUR; A, affordable; S, sensitivity; S, specificity; U, Number of user steps; R, rapid turnaround time; INF, influenza A; MS2, male specific bacteriophage; MTB, *M*. *tuberculosis*; SPN, *S*. *pneumoniae*; STM, *S*. Typhimurium.

The goal of this study was to identify NA extraction technologies that fit the requirements for integration into a test device for the molecular detection of infectious agents. We already described the analytical performance in terms of sensitivity and specificity of RT-PCR based detection from the NA extracts as compared to reference methods using the MagNaPure (CDC) or Qiagen kits (PATH; Table 2). While important, analytical performance is not the only metric that should be considered when ranking the technologies. We also applied the ASSUR criteria defined in the Results section in a series of Radar plots to visually compare each technology with each sample type assessed for both target microbes (Fig 2). The normalized parameters in each panel were average by panel and also overall to permit ranking of each technology in comparison to simply performance (Table 4; data file at https://dataverse.harvard.edu/dataverse/SSB).

**Table 4:** The ranking of each developers based on analyses of radial plot values. The five data points for each ASSUR radial data point were equally weighted and the averaged data within each plot was calculated as compared to optimal ASSUR plot values of 1.0 (Fig 2). The developers were ranked for each of the three sample panels and overall via the average of the pooled values for both targets per sample panel and for all six targets for overall. The developers were ranked in ascending order. The ranking of the developers based upon sensitivity and specificity only is provided in parentheses; FLU, influenza A, MTB, *M*. *tuberculosis*; SP, sputum; MS2, male specific bacteriophage; SPN, *S*. *pneumoniae*; BL, blood, STM, *S*. Typhimurium; ST, stool.

Developer	FLU	MTB	SP rank	MS2	SPN	BL rank	MS2	STM	ST rank	Overall rank
A	0.65	0.67	4 (3)	0.68	0.68	3 (1)	0.61	0.64	3 (1)	**2 (1)**
B	0.54	0.62	5 (5)	0.61	0.71	4 (6)	0.74	0.69	2 (6)	**3 = (6)**
C	0.80	0.78	1 (1)	0.68	0.75	2 (4)	0.63	0.56	4 (4)	**3 = (4)**
D	0.44	0.49	6 (6)	0.50	0.61	6 (5)	0.59	0.60	5 (5)	**6 (5)**
E	0.72	0.72	3 (2)	0.65	0.66	5 (3)	0.59	0.57	6 (3)	**3 = (3)**
F	0.75	0.75	2 (4)	0.80	0.82	1 (2)	0.76	0.74	1 (2)	**1 (2)**

The ASSUR rankings (Table 4) were compared to the performance ranking displayed in Table 2. Developer F replaced A as the overall highest ranked group with changes of one or two places for the other developers. With the sputum panel, developer C retained the highest rank and developer F was second; developers A and E dropped one place from the original ranking (to 4^th^ and 3^rd^ respectively) while B and D remained the same. The blood panel ASSUR ranking changed for each developer as compared to performance ranking with developers F and C scoring the top rankings, respectively. The ranking of developers A and E each dropped by two places to third and fifth whilst developer B scored fourth and D dropped to sixth. In ranking the stool averages developer F was again ranked highest with developer B ranked as second. This result represented the only significant increase of more than two ranking places in any of the panels. Developers both dropped in the rankings to third and sixth respectively with the reaming developers retaining the same rank.

The addition of data to further inform on the suitability of the technologies to be incorporated into a low cost NAAT resulted in slight changes to the overall ranking of technologies as compared to performance only, though both ranking algorithms identified developers A and F as the top two technologies developed by Akonni Biosystems and Molbio Diagnostics respectively. The inclusion of turnaround times (TAT) and manual steps into the ASSUR dataset were the underlying reasons for Molbio (F) moving ahead of Akonni Biosystems (A) A. Molbio also had good performance but also a more rapid TAT (35 minutes versus 55 minutes) and fewer user steps than the Akonni methodology and so was awarded higher ranking via the radar plot analyses (Fig 1). Both of these developers use systems that are automated to differing degrees with the Molbio platform hosting a cassette that processes the sample preparation steps [33,34]. The data from other developers such as B also improved, primarily due to the incorporation of the fastest TATs to augment their relatively poor performance data (Table 2 and Fig 1). In light of these findings, the technologies offered by Akonni and Molbio have the best scores for consideration to enter the next stage of assessment for integration into a fully automated stand-alone NAAT platform.

## Discussion

In this study we report on an initial screen of six candidate NA extraction technologies assessing their suitability for integration into a low cost NAAT cartridge with performance comparisons to two established NA extraction systems offered by the Roche MagNaPure (automated) and Qiagen (manual) using contrived panels of samples that mimic common clinical specimens. These technologies are in various phases of product development, ranging from alpha prototypes to products that are on or close to market (e.g. Akonni and Molbio). Therefore, poorer performance may simply reflect that the devices or methods are still being refined. Several of the developers also noted that they normally use different protocols and materials for extracting RNA or DNA, and thus had to make some modifications to co-extract both DNA and RNA to meet a requirement of a potential integrated NAAT. Consequently, some technologies may have been suboptimal under the conditions imposed for this study. We investigated the diagnostic performance and workflow of these technologies by challenging them with three panels of contrived samples representing common specimen types used in the NAAT-based diagnosis of multiple pathogens: 1) sputum (tuberculosis); 2) stool (diarrheal diseases, e.g. *Shigella* spp.); and 3) blood (e.g. HIV, malaria, and typhoid). Target microbes were spiked at different concentrations to assess the effectiveness of the different NA extraction methods across a range of target burdens within a complex matrix. In each panel, two spiked microbes were selected to evaluate the efficiency of extraction from both DNA- and RNA-based genomic targets. While the reference methods typically gave the best results, the Akonni platform had better performance with certain target pathogens. Akonni is heavily invested in MTB diagnostic NAATs and so their best in class performance for MTB likely reflects their expertise in this area [35–37].

The specimen type and associated microbes were chosen to present different challenges for NA extraction in terms of the composition of the specimen matrix [20–25,38,39], matrix associated RT-PCR confounders [21], and the load and structural complexity of target microbes (e.g. acid fast, gram positive/negative bacteria and RNA-base viruses). To minimize bias and optimize extraction performance, each developer was provided with identical blinded sample panels that were processed at their facilities, using their equipment operated by trained staff. The NA extracts from each group were subsequently screened at a national reference laboratory with validated RT-PCR assays that used the same platform, reagents, users and protocols.

The six technologies were ranked in this exercise with an emphasis on extraction and detection efficacy of target NAs from complex specimen matrices. Two methods were used to score the developers relative to each other. One was based solely on performance (i.e. sensitivity and specificity) and the other included the turnaround time, number of manual steps and the estimated cost per extraction. Based on performance, Akonni (developer A) ranked highest with five of the six targets used in the three specimen panels. The Akonni platform is partially automated and utilizes their TruTip technology; a silica coated frit housed in a 1 mL micropipettor tip [37]. Negative and positive air pressure is used to manipulate liquid lysed material and buffers across the frit to sequentially capture, wash and elute NAs from a sample using an automated pipettor [33,40]. Molbio and developer E ranked second and third, respectively, but did not consistently detect all NA replicates in the lowest spiked samples. The Molbio platform is the Trueprep AUTO, a battery powered instrument that hosts a cartridge-based extraction system that utilizes Boom chemistry to capture and purify NAs [41].

Observed specificity for most methods was relatively uniform among five of the six technologies but only one developer (C) scored 100%. As with any laboratory procedure, it is possible that contamination of specimens or extracts occurred during preparation, processing, handling, or testing, as observed with MS2 in extracts from developer B. The risk of operator-introduced contamination is lower when automated systems are used and was observed with the both Akonni and Molbio platforms that have varying degrees of automation [33,34]. Good laboratory practices for molecular testing laboratories were followed by all laboratories to minimize the potential for contamination of specimens, extracts, or PCR reactions in order to ensure integrity of results.

The incorporation of additional metrics related to user input, TAT, and ease of use, to assess the developers resulted in slight changes to the ranking. Molbio was ranked highest with Akonni being second. Other developers that ranked lower on performance only scored significantly better when these other factors were included. This is in part due to equal weighting of the five metrics used. Some developers claimed to focus on performance with extended processing times as compared to rapid processing times or limiting the number of user steps. It is feasible that with protocol refinement and automation these challenges can be addressed as the ideal test operation must have minimal or no user inputs. Costing was biased in that all developers reported that US$2 per extraction was feasible yet none are either in production or manufacturing at a scale that can confirm accurately meeting this price.

There are several limitations to the methods used in this head-to-head study. Firstly, because the stool and blood used to generate contrived samples were acquired from single individuals, these may not represent the often challenging heterogeneity seen with samples from different patients. Further, the composition of sputum and typically stool from healthy donors is not representative of the same matrices during the course of an active infection (e.g. presence of blood). Although the RNA and DNA-based microbes used for spiking provided a standardized means to assess differences in extraction efficiency of the various technologies, some targets, such as the chemically inactivated *M*. *tuberculosis* cells, may not represent the same challenge to cell lysis as live mycobacteria. Similarly, *S*. *pneumoniae* is prone to autolysis when cultured to late growth phase and so the cells prepared for spiking may have been partially compromised during their culture, harvesting or storage. This study was focused on NA extraction efficiency in terms of detecting specific target NAs but did not address matrix effects associated with potential inhibitory compounds that could affect the performance of the RT-PCR reactions [20,22,26]. It is feasible that some low ranked methods were highly efficient at extracting NAs but an associated co-purification of confounders masked the former by inhibiting amplification by RT-PCR. While variation in C_t_ values was used to semi-quantitatively assess NA extraction efficiencies, the wide range of sample input and elution volumes used by the different methods makes it difficult to directly compare and normalize extraction efficiencies.

The intent of this study was to assess different NA extraction tools to identify best in class candidates with potential for integration into an affordable, automated test cartridge that offers high diagnostic accuracy when combined with a suitable amplification assay. The Akonni Biosystems and Molbio ranked best via either scoring method. Both companies have NA extraction products on the market and so this exercise highlights a basic fact that products validated for release onto the market typically have better performance than a prototype that is still in development. Several key elements necessary to meet this goal were beyond the scope of this study, including design for manufacturing. While multiple features can be integrated into a single test cartridge, increased design complexity typically raises manufacturing costs and/or failure modes. One area of future investigation is to optimize for the maximum volume of eluate for amplification and detection such that the eluate or an aliquot thereof is the only liquid necessary to add to lyophilized amplification reagents. A parallel evaluation of suitable candidates for NA amplification was recently completed and is presented in a companion manuscript [42]. Ultimately, we intend to assess the most suitable methods for both NA extraction and amplification/detection for integration and potentially enable the development of a low-cost point of care NAAT that meets the clinical and economic challenges for use in RLS.

## References

[1] Drain PK, Hyle EP, Noubary F, Freedberg KA, Wilson D, Bishai WR, et al Diagnostic point-of-care tests in resource-limited settings. Lancet Infect Dis. 2014;14: 239–249. 10.1016/S1473-3099(13)70250-0 24332389 PMC4016042

[2] Halse TA, Edwards J, Cunningham PL, Wolfgang WJ, Dumas NB, Escuyer VE, et al Combined real-time PCR and rpoB gene pyrosequencing for rapid identification of Mycobacterium tuberculosis and determination of rifampin resistance directly in clinical specimens. J Clin Microbiol. 2010;48: 1182–1188. 10.1128/JCM.02149-09 20107097 PMC2849603

[3] Santos CG, Sabido M, Leturiondo AL, Ferreira CO, da Cruz TP, Benzaken AS. Development, validation and testing costs of an in-house real-time PCR assay for the detection of Chlamydia trachomatis. J Med Microbiol. 2017;66: 312–317. 10.1099/jmm.0.000443 28141509

[4] Niemz A, Ferguson TM, Boyle DS. Point-of-care nucleic acid testing for infectious diseases. Trends Biotechnol. 2011;29: 240–250. 10.1016/j.tibtech.2011.01.007 21377748 PMC3746968

[5] Denkinger CM, Kik SV, Pai M. Robust, reliable and resilient: designing molecular tuberculosis tests for microscopy centers in developing countries. Expert Rev Mol Diagn. 2013;13: 763–767. 10.1586/14737159.2013.850034 24151844

[6] Weigl BH, Boyle DS, de los ST, Peck RB, Steele MS. Simplicity of use: a critical feature for widespread adoption of diagnostic technologies in low-resource settings. Expert Rev Med Devices. 2009;6: 461–464. 10.1586/erd.09.31 19751117

[7] Mateen FJ, McKenzie ED, Rose S. Medical Schools in Fragile States: Implications for Delivery of Care. Health Serv Res. 2018;53: 1335–1348. 10.1111/1475-6773.12709 29368334 PMC5980181

[8] Schmidt BM, Geldenhuys H, Tameris M, Luabeya A, Mulenga H, Bunyasi E, et al Impact of Xpert MTB/RIF rollout on management of tuberculosis in a South African community. S Afr Med J. 2017;107: 1078–1081. 10.7196/SAMJ.2017.v107i12.12502 29262960

[9] Denkinger CM, Nicolau I, Ramsay A, Chedore P, Pai M. Are peripheral microscopy centres ready for next generation molecular tuberculosis diagnostics? Eur Respir J. 2013;42: 544–547. 10.1183/09031936.00081113 23904551

[10] Bwirire LD, Fitzgerald M, Zachariah R, Chikafa V, Massaquoi M, Moens M, et al Reasons for loss to follow-up among mothers registered in a prevention-of-mother-to-child transmission program in rural Malawi. Trans R Soc Trop Med Hyg. 2008;102: 1195–1200. 10.1016/j.trstmh.2008.04.002 18485431

[11] MacPherson P, MacPherson EE, Mwale D, Bertel SS, Makombe SD, Corbett EL, et al Barriers and facilitators to linkage to ART in primary care: a qualitative study of patients and providers in Blantyre, Malawi. J Int AIDS Soc. 2012;15: 18020 10.7448/IAS.15.2.18020 23336700 PMC3535694

[12] Hanrahan CF, Selibas K, Deery CB, Dansey H, Clouse K, Bassett J, et al Time to treatment and patient outcomes among TB suspects screened by a single point-of-care xpert MTB/RIF at a primary care clinic in Johannesburg, South Africa. PLoS One. 2013;8: e65421 10.1371/journal.pone.0065421 23762367 PMC3675091

[13] Mwansa-Kambafwile J, Maitshotlo B, Black A. Microbiologically Confirmed Tuberculosis: Factors Associated with Pre-Treatment Loss to Follow-Up, and Time to Treatment Initiation. PLoS One. 2017;12: e0168659 10.1371/journal.pone.0168659 28068347 PMC5222612

[14] Cohen GM, Drain PK, Noubary F, Cloete C, Bassett IV. Diagnostic delays and clinical decision making with centralized Xpert MTB/RIF testing in Durban, South Africa. J Acquir Immune Defic Syndr. 2014;67: e88–e93. 10.1097/QAI.0000000000000309 25314255 PMC4197409

[15] Naidoo P, Dunbar R, du TE, van NM, Squire SB, Beyers N, et al Comparing laboratory costs of smear/culture and Xpert(R) MTB/RIF-based tuberculosis diagnostic algorithms. Int J Tuberc Lung Dis. 2016;20: 1377–1385. 10.5588/ijtld.16.0081 27725051

[16] World Health Organization. High-priority target product profiles for new tuberculosis diagnostics: report of a consensus meeting. Geneva: World Health Organization; 2014. http://apps.who.int/iris/bitstream/10665/135617/1/WHO_HTM_TB_2014.18_eng.pdf

[17] MouraI A, Hodon MA, Soares FilhoI PM, de Azevedo IssaI M, Ferreira de Oliveira AP, Fonmseca AA. Comparison of nine DNA extraction methods for the diagnosis of bovine tuberculosis by real time PCR. Ciência Rural. 2016;46: 1223–1228.

[18] Paulos S, Mateo M, de LA, Hernandez-de MM, Bailo B, Saugar JM, et al Evaluation of five commercial methods for the extraction and purification of DNA from human faecal samples for downstream molecular detection of the enteric protozoan parasites Cryptosporidium spp., Giardia duodenalis, and Entamoeba spp. J Microbiol Methods. 2016;127: 68–73. 10.1016/j.mimet.2016.05.020 27241828

[19] Petrich A, Mahony J, Chong S, Broukhanski G, Gharabaghi F, Johnson G, et al Multicenter comparison of nucleic acid extraction methods for detection of severe acute respiratory syndrome coronavirus RNA in stool specimens. J Clin Microbiol. 2006;44: 2681–2688. 10.1128/JCM.02460-05 16891478 PMC1594626

[20] Al-Soud WA, Radstrom P. Purification and characterization of PCR-inhibitory components in blood cells. J Clin Microbiol. 2001;39: 485–493. 10.1128/JCM.39.2.485-493.2001 11158094 PMC87763

[21] Garcia ME, Blanco JL, Caballero J, Gargallo-Viola D. Anticoagulants interfere with PCR used to diagnose invasive aspergillosis. J Clin Microbiol. 2002;40: 1567–1568. 10.1128/JCM.40.4.1567-1568.2002 11923400 PMC140326

[22] Monteiro L, Bonnemaison D, Vekris A, Petry KG, Bonnet J, Vidal R, et al Complex polysaccharides as PCR inhibitors in feces: Helicobacter pylori model. J Clin Microbiol. 1997;35: 995–998. 9157172 10.1128/jcm.35.4.995-998.1997 PMC229720

[23] Rohrman B, Richards-Kortum R. Inhibition of recombinase polymerase amplification by background DNA: a lateral flow-based method for enriching target DNA. Anal Chem. 2015;87: 1963–1967. 10.1021/ac504365v 25560368

[24] Schrader C, Schielke A, Ellerbroek L, Johne R. PCR inhibitors—occurrence, properties and removal. J Appl Microbiol. 2012;113: 1014–1026. 10.1111/j.1365-2672.2012.05384.x 22747964

[25] Sidstedt M, Hedman J, Romsos EL, Waitara L, Wadso L, Steffen CR, et al Inhibition mechanisms of hemoglobin, immunoglobulin G, and whole blood in digital and real-time PCR. Anal Bioanal Chem. 2018;410: 2569–2583. 10.1007/s00216-018-0931-z 29504082 PMC5857286

[26] Voynow JA, Rubin BK. Mucins, mucus, and sputum. Chest. 2009;135: 505–512. 10.1378/chest.08-0412 19201713

[27] Dong M, Fisher C, Anez G, Rios M, Nakhasi HL, Hobson JP, et al Standardized methods to generate mock (spiked) clinical specimens by spiking blood or plasma with cultured pathogens. J Appl Microbiol. 2016;120: 1119–1129. 10.1111/jam.13082 26835651 PMC4811715

[28] Scott LE, Gous N, Cunningham BE, Kana BD, Perovic O, Erasmus L, et al Dried culture spots for Xpert MTB/RIF external quality assessment: results of a phase 1 pilot study in South Africa. J Clin Microbiol. 2011;49: 4356–4360. 10.1128/JCM.05167-11 21976767 PMC3232964

[29] United States Environmental Protection Agency. Method 1601: Male-specific (F+) and Somatic Coliphage in Water by Two-step Enrichment Procedure. Washington, DC.: United States Environmental Protection Agency; 2001. https://www.epa.gov/sites/production/files/2015-12/documents/method_1601_2001.pdf

[30] Vivas-Alegre S, Fernandez-Natal I, Lopez-Fidalgo E, Rivero-Lezcano OM. Preparation of inocula for experimental infection of blood with Streptococcus pneumoniae. MethodsX. 2015;2: 463–468. 10.1016/j.mex.2015.11.003 26844211 PMC4688400

[31] Perez-Osorio AC, Boyle DS, Ingham ZK, Ostash A, Gautom RK, Colombel C, et al Rapid identification of mycobacteria and drug-resistant Mycobacterium tuberculosis by use of a single multiplex PCR and DNA sequencing. J Clin Microbiol. 2012;50: 326–336. 10.1128/JCM.05570-11 22162548 PMC3264146

[32] Peeling RW, Holmes KK, Mabey D, Ronald A. Rapid tests for sexually transmitted infections (STIs): the way forward. Sex Transm Infect. 2006;82 Suppl 5: v1–v6. 17151023 10.1136/sti.2006.024265 PMC2563912

[33] Thakore N, Garber S, Bueno A, Qu P, Norville R, Villanueva M, et al A bench-top automated workstation for nucleic acid isolation from clinical sample types. J Microbiol Methods. 2018;148: 174–180. 10.1016/j.mimet.2018.03.021 29678500 PMC5944857

[34] Nikam C, Jagannath M, Narayanan MM, Ramanabhiraman V, Kazi M, Shetty A, et al Rapid diagnosis of Mycobacterium tuberculosis with Truenat MTB: a near-care approach. PLoS One. 2013;8: e51121 10.1371/journal.pone.0051121 23349670 PMC3549918

[35] Linger Y, Kukhtin A, Golova J, Perov A, Qu P, Knickerbocker C, et al Demonstrating a multi-drug resistant Mycobacterium tuberculosis amplification microarray. J Vis Exp. 2014; 10.3791/51256 PMC4027031 24796567

[36] Linger Y, Knickerbocker C, Sipes D, Golova J, Franke M, Calderon R, et al Genotyping Multidrug-Resistant Mycobacterium tuberculosis from Primary Sputum and Decontaminated Sediment with an Integrated Microfluidic Amplification Microarray Test. J Clin Microbiol. 2018;56:10.1128/JCM.01652-17 PMC5824040 29305543

[37] Thakore N, Norville R, Franke M, Calderon R, Lecca L, Villanueva M, et al Automated TruTip nucleic acid extraction and purification from raw sputum. PLoS One. 2018;13: e0199869 10.1371/journal.pone.0199869 29975759 PMC6033430

[38] Al-Soud WA, Jonsson LJ, Radstrom P. Identification and characterization of immunoglobulin G in blood as a major inhibitor of diagnostic PCR. J Clin Microbiol. 2000;38: 345–350. 10618113 10.1128/jcm.38.1.345-350.2000 PMC88721

[39] Thornton CG, Passen S. Inhibition of PCR amplification by phytic acid, and treatment of bovine fecal specimens with phytase to reduce inhibition. J Microbiol Methods. 2004;59: 43–52. 10.1016/j.mimet.2004.06.001 15325752

[40] Holmberg RC, Gindlesperger A, Stokes T, Lopez D, Hyman L, Freed M, et al Akonni TruTip((R)) and Qiagen((R)) methods for extraction of fetal circulating DNA—evaluation by real-time and digital PCR. PLoS One. 2013;8: e73068 10.1371/journal.pone.0073068 23936545 PMC3735556

[41] Nair CB, Manjula J, Subramani PA, Nagendrappa PB, Manoj MN, Malpani S, et al Differential Diagnosis of Malaria on Truelab Uno(R), a Portable, Real-Time, MicroPCR Device for Point-Of-Care Applications. PLoS One. 2016;11: e0146961 10.1371/journal.pone.0146961 26784111 PMC4718663

[42] Cantera J, White H, Diaz MH, Beall SG, Winchell JM, Lillis L, et al The assessment of eight nucleic acid amplification technologies for the molecular detection of infectious agents in low-resource settings. PLoS One. 2019; 10.1371/journal.pone.0215756 PMC6476514 31009510

